# Study on the electrodeposition of uranium in chloride molten salt

**DOI:** 10.1039/d4ra00607k

**Published:** 2024-02-27

**Authors:** Pei Wu, Liqin Wang, Jinrui Wang, Junhan Luo, Yuexiang Lu, Xiaopeng Song, Jilian Liu, Yongquan Qin, Liudong Hou, Jing Ma

**Affiliations:** a China Nuclear Power Engineering Co., Ltd. Beijing 100840 People's Republic of China 13671212490@163.com; b Institute of Nuclear and New Energy Technology, Tsinghua University Beijing 100084 People's Republic of China

## Abstract

This study focuses on the recovery of UO_2_ from oxide spent fuel using electrodeposition. U_3_O_8_ was used as the initial material and dissolved in NaCl–2CsCl using NH_4_Cl at high temperatures by means of chlorination reaction. The electrolysis process was conducted using a three-electrode system to investigate the effects of cathode material and diameter, electrolysis temperature, electrolysis time, electrolysis voltage, and uranium concentration in the molten salt on the electrolysis reaction. By optimizing the electrolysis conditions, pure UO_2_ with a recovery efficiency of 97% was obtained, and the products were characterized using XRD, SEM-EDS, ICP-AES and XPS. It was found that within the scope of this experiment, increasing the cathode diameter, extending the electrolysis time, and increasing the reduction voltage appropriately all led to an improvement in the recovery efficiency of the electrolysis reaction, while other conditions had minimal effect on the reaction. Furthermore, doping of the electrolyte system was performed by adding La, Ce and Nd elements, while the removal of La showed good purification effects, with a maximum decontamination factor of 119. Furthermore, the system showed good purification effects for Nd, with a decontamination factor of 57.

## Abstract 2

Pure UO_2_ was obtained with a recovery efficiency up to 97%. The system exhibited better removal of La with a decontamination factor as high as 119.

## Graphical abstract

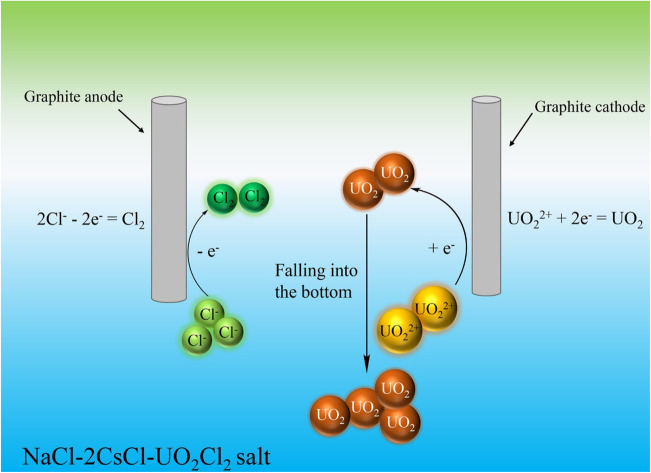

## 1 Introduction

Nuclear energy is an important clean energy source that can effectively replace fossil fuel power generation, thereby reducing greenhouse gas emissions. In the current stage of sustainable development, the main issues related to nuclear energy include how to maximize the utilization of uranium resources and how to minimize nuclear waste.^1^ Dry reprocessing for spent fuel, based on molten salt electrolysis, offers unique advantages such as small-scale operations, low waste generation, high radiation tolerance, a simple process, and effective prevention of nuclear proliferation.^2^ It is currently considered one of the most effective methods for spent fuel reprocessing.

Dry reprocessing of spent fuel is one of the critical steps in the nuclear fuel cycle. In recent years, dry reprocessing based on molten salt electrolysis has been considered one of the most effective methods for spent fuel reprocessing due to its unique advantages. The United States has developed molten salt electrorefining technology for spent metal fuel, while Russia has developed molten salt electrodeposition technology for metal oxide spent fuel. The dry reprocessing for oxide spent fuel based on molten salt system electrodeposition technology has a high maturity.^3^ In comparison to electrolytic refining in molten salt, the electrodeposition process is simpler, and the prepared mixed oxide of uranium and plutonium can be applied in the MOX of fast reactors.

Due to the high efficiency, conductivity, and selectivity, molten salt electrolysis method has become a commonly method for depositing metals, purifying metals and synthesizing functional materials in recent years.^4–9^ In the electrochemical deposition process, the dissolution and chlorination of oxides are key steps. Effectively chlorinating uranium oxide compounds in molten salt and reducing its corrosiveness to the device remains a significant challenge. For example, the Russian Research Institute of Atomic Reactors (RIAR) used Cl_2_ as a chlorinating agent,^10^ which generates a large amount of toxic and harmful exhaust gases. On the other hand, Cl_2_ as a chlorinating agent, can generate highly volatile UCl_5_ and UCl_6_ at high temperatures, which may lead to partial loss of uranium.^11^ UO_2_ exhibits low solubility in molten MgCl_2_ or CaCl_2_.^12^ Thermodynamic estimates suggest that the chlorination of UO_2_ is difficult in the ZrCl_4_–LiCl–KCl molten salt system.^13^ Accordingly, the aim of this experiment was to explore the recovery efficiency of UO_2_ from spent oxide fuel. First, UO_2_ was oxidized to U_3_O_8_ in an air environment.

Compared to NaCl–LiCl and NaCl–KCl eutectic salt, NaCl–2CsCl has some advantages of higher solubility for Cl_2_ and a lower melting point.^4,14^ Therefore, NaCl–2CsCl was chosen as reaction medium in the electrodeposition process. Additionally, the optimal parameters obtained from this work can be applied to the engineering field of scale expansion in the future. Considering the aspect of engineering applications, U_3_O_8_ obtained from spent fuel after high-temperature oxidation, and the dissolution of U_3_O_8_ was faster than that of UO_2_ under the air atmosphere.^15^ Therefore, U_3_O_8_ was used as a reactant to react with NH_4_Cl to generate UO_2_Cl_2_.^11^ Currently, this method proves to be effective for dissolving and chlorinating uranium oxide compounds. After cooling, the mixture was transferred to a glove box for electrolysis, leading to the production of UO_2_. Herrmann conducted electrolytic reduction refining on oxide spent fuel to recover metal U,^16^ and the decontamination factors for neodymium (Nd), cerium (Ce), and lanthanum (La) were only 25, 11, and 24.^17^ Considering the low decontamination factors of La, Ce and Nd by electrolytic reduction refining method, combining with the higher content of elements in real spent fuel, La, Ce and Nd were selected as doping elements for electrochemical deposition. The purity and morphology of UO_2_ in the product were analyzed using characterization techniques such as X-ray Diffraction (XRD), Inductively Coupled Plasma Atomic Emission Spectroscopy (ICP-AES), Scanning Electron Microscopy-Energy Dispersive X-ray Spectroscopy (SEM-EDS) and X-ray Photoelectron Spectroscopy (XPS). The recovery efficiency and decontamination factor were calculated based on the initial addition weight of U_3_O_8_ and impurity elements, laying the foundation for the industrial implementation of dry processing for depleted fuel.

## 2 Results and discussion

### 2.1 Oxidation reaction of UO_2_

Heating 9.9 g of UO_2_ at 650 °C for 5.5 h can product 10.0 g of U_3_O_8_ in a yield of 96.3%, as shown in eqn (1).
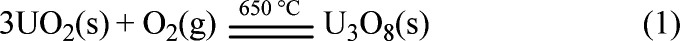


**Fig. 1:**
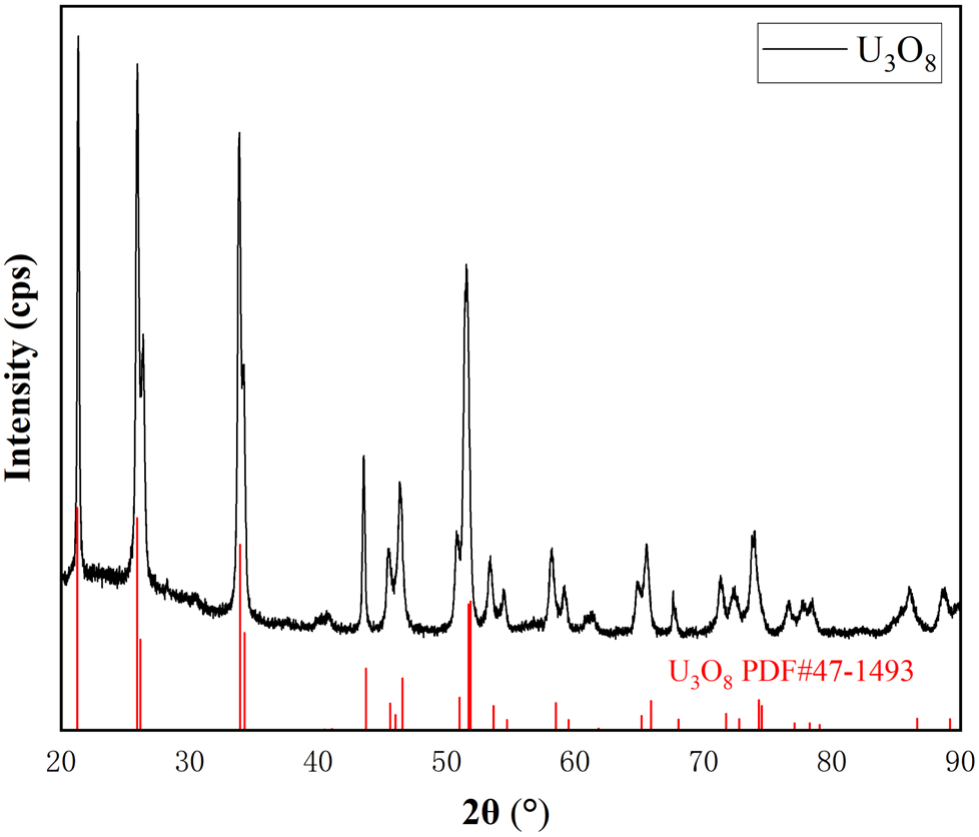
XRD of oxidation product U_3_O_8_.

As shown in Fig. 1, the peaks in the XRD spectrum correspond to the standard card of U_3_O_8_, and there are no other peaks, indicating that UO_2_ has been completely oxidized to pure U_3_O_8_.

### 2.2 Chlorination reaction of UO_2_ (ref. 11 and 15)

**Fig. 2:**
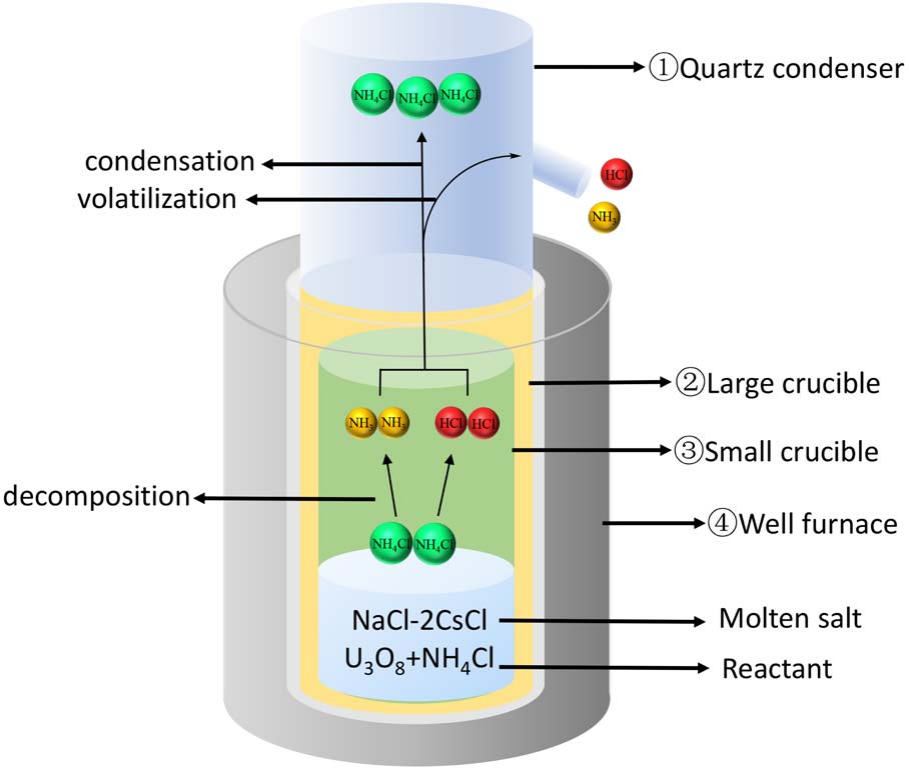
Chlorination device for U_3_O_8_.

Using NH_4_Cl to chlorinate U_3_O_8_ to generate UO_2_Cl_2_, as shown in Fig. 2. Firstly, placed the large crucible ② (which protects the small crucible ③) into a high-temperature well furnace ④ and heated it to 650 °C in a fume hood. Mixed the prepared molten salt, U_3_O_8_, and NH_4_Cl evenly and added them to the small crucible ③. Then, placed the small crucible ③ into the large crucible ②, and installed a quartz condenser cover ① above it. Obviously, O_2_ in the air played an important role in the dissolution process, as it transformed tetravalent uranium into a higher valence state, which was the decisive factor in the formation of UO_2_Cl_2_.^11,18^ The reaction is shown in eqn (2).
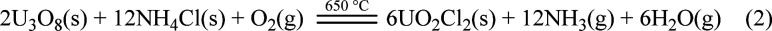


### 2.3 Optimization of electrodeposition reaction conditions

The Cl^−^ in the chloride molten salt undergoes an oxidation reaction at the anode, accompanied by the formation of Cl_2_. While UO_2_^2+^ gains electrons at the cathode and generates UO_2_. Different cathode has different reduction processes for UO_2_^2+^.^19^ When W^20–22^ and glassy carbon^23^ were used, the reaction took two steps, and the formation of intermediate product UO_2_^+^ was found in the molten salt, which was unstable and easily disproportionated into UO_2_ and UO_2_^2+^, while SnO_2_,^24^ Pt,^25^ graphite^26^ could reduce UO_2_^2+^ to UO_2_ directly.

In this experiment, the electrode material and diameter, electrolysis time, temperature, uranium concentration in molten salt, and electrolysis voltage were selected to investigate the influence of different conditions of electrodeposition reaction on the recovery efficiency of UO_2_.

Firstly, dissolved 0.1 g U_3_O_8_ in 10 g of molten salt (*n*(NaCl) : *n*(CsCl) = 1 : 2) at 650 °C, by means of *Φ* 6 mm graphite as anode and *Φ* 1 mm platinum as cathode, *Φ* 0.5 mm platinum was used as the reference electrode. Based on the reported Cyclic Voltammetry (CV) curves of UO_2_Cl_2_,^11^ the initial electrolysis voltage was selected as −1.2 V. Additionally, electrolyzed the U_3_O_8_ for 2 h at 650 °C in the argon glove box, which can act as the initial condition. After washing, centrifugation, and drying, pure UO_2_ was obtained with a recovery efficiency of 70%.3
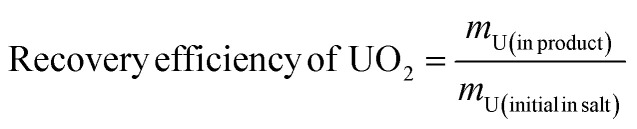


#### 2.3.1 Optimization of electrode specifications

Firstly, the cathode material diameter was optimized. When *Φ* 2 mm platinum was used as the cathode, the recovery efficiency increased to 90%, indicating that the electrodeposition effect of *Φ* 2 mm platinum is better than that of *Φ* 1 mm platinum, this may be due to the larger specific surface of *Φ* 2 mm platinum, decreasing the current density. Subsequently, *Φ* 5 mm graphite was used for testing, and the recovery efficiency of UO_2_ was similar to the result obtained with the *Φ* 2 mm platinum. Considering potential experimental errors in operation and analysis, the cost-effective *Φ* 5 mm graphite with nearly the same recovery efficiency was chosen as the cathode (Table 1).

**Table 1:** The effect of cathode materials on electrodeposition reactions

No.	Cathode material and diameter (mm)	Temperature (°C)	Electrolysis time (h)	Uranium concentration in molten salt (U_3_O_8_ : molten salt)	Electrolysis voltage (V)	Recovery efficiency of UO_2_ (%)
1	*Platinum Φ 1*	650	2	1 : 100	−1.2	70
2	*Platinum Φ 2*	650	2	1 : 100	−1.2	90
**3**	***Graphite Φ 5***	**650**	**2**	**1** **:** **100**	**−1.2**	**89**

#### 2.3.2 Optimization of electrolysis time

As shown in Table 2, when the reaction time was extended from 2 h to 5 h, the recovery efficiency of UO_2_ gradually increased. Further extending the time to 6 h did not result in a significant change in the recovery efficiency, it can illustrate that after 5 h of reaction, the concentration of U in the molten salt has exhausted. Therefore, it can be observed that prolonging the reaction time appropriately can improve the recovery efficiency of UO_2_. Therefore, the optimal condition for the electrolysis time was determined to be 5 h.

**Table 2:** The effect of electrolysis time on electrodeposition reaction

No.	Cathode material and diameter (mm)	Temperature (°C)	Electrolysis time (h)	Uranium concentration in molten salt (U_3_O_8_ : molten salt)	Electrolysis voltage (V)	Recovery efficiency of UO_2_ (%)
3	Graphite *Φ* 5	650	*2*	1 : 100	−1.2	89
4	Graphite *Φ* 5	650	*3*	1 : 100	−1.2	92
**5**	**Graphite *Φ* 5**	**650**	*5*	**1** : **100**	**−1.2**	**95**
6	Graphite *Φ* 5	650	*6*	1 : 100	−1.2	95

#### 2.3.3 Optimization of electrolysis temperature

RIAR found that uranium dioxide with good crystallinity can be obtained at a temperature of 600–650 °C.^27^ When the electrolysis temperature was lowered from 650 °C to 600 °C or raised to 700 °C, there was no significant change in the recovery efficiency of UO_2_, which remained at approximately 93–95%. Considering that higher temperature increases the corrosiveness of the electrolysis equipment, and based on the recovery efficiency obtained from the experiments, the optimal electrolysis temperature was determined to be 650 °C (Table 3).

**Table 3:** The effect of electrolysis temperature on electrodeposition reaction

No.	Cathode material and diameter (mm)	Temperature (°C)	Electrolysis time (h)	Uranium concentration in molten salt (U_3_O_8_ : molten salt)	Electrolysis voltage (V)	Recovery efficiency of UO_2_ (%)
7	Graphite *Φ* 5	*600*	5	1 : 100	−1.2	94
**5**	**Graphite *Φ* 5**	***650***	**5**	**1** : **100**	**−1.2**	**95**
8	Graphite *Φ* 5	*700*	5	1 : 100	−1.2	93

#### 2.3.4 Optimization of uranium concentration

By changing the initial concentration of U_3_O_8_ in the molten salt, the influence of uranium concentration on the electrodeposition reaction was investigated. As shown in Table 4, 0.2 g U_3_O_8_ was dissolved in 10 g mixed molten salt (NaCl–2CsCl), with other conditions kept constant, the recovery efficiency of UO_2_ showed a high value of 96% after electrodeposition. Meanwhile, by decreasing the concentration of U_3_O_8_ and dissolving 0.1 g of U_3_O_8_ in 20 g mixed molten salt, a recovery efficiency of 94% was achieved, while the proportion of molten salt components remained unchanged. Considering that increasing the amount of uranium used would decrease the impact on the recovery efficiency due to uranium loss during the experimental process, and there is no significant change in the recovery efficiency of uranium in fact, the mass ratio of 1 : 100 between U_3_O_8_ and the molten salt was chosen as the optimal concentration. This ensures a reduction in uranium usage as much as possible while maintaining ease of operation.

**Table 4:** The effect of uranium concentration on electrodeposition reaction

No.	Cathode material and diameter (mm)	Temperature (°C)	Electrolysis time (h)	Uranium concentration in molten salt (U_3_O_8_ : molten salt)	Electrolysis voltage (V)	Recovery efficiency of UO_2_ (%)
9	Graphite *Φ* 5	650	5	*1* *:* *50*	−1.2	96
**5**	**Graphite *Φ* 5**	**650**	**5**	***1*** *:* ***100***	**−1.2**	**95**
10	Graphite *Φ* 5	650	5	*1* *:* *200*	−1.2	94

#### 2.3.5 Optimization of electrolysis voltage

To investigate the influence of electrolysis voltage on the electrodeposition reaction, the voltage was adjusted to −1.6 V and −0.8 V. When the voltage was set at −1.6 V, the recovery efficiency of UO_2_ was 97%. When the reduction voltage was −0.8 V, low applied voltage leads to a decrease in current during the electrolysis process. As the number of electrons transferred per unit time decreases, the electrolysis rate will decrease, resulting in a higher current generated after 5 h of electrolysis. Therefore, the electrolysis time was extended to 9 h, resulting in a recovery efficiency of 91%. By adjusting the electrolysis voltage, it was found that reducing the reduction voltage would decrease the rate of electrodeposition, which is unfavourable for the progression of the reaction. Although increasing the reduction voltage would slightly improve the recovery efficiency of UO_2_, excessive higher reduction voltages may cause side reactions, leading to the reduction of impurity ions and mix them into the UO_2_ product. Hence, the electrolysis voltage should be set based on the actual reduction voltage of the doping elements present.

### 2.4 Characterization of UO_2_

Factors such as cathode material and electrolysis voltage can influence the morphology and composition of the deposition. In this experiment, XRD, SEM-EDS, ICP-AES and XPS characterization were performed on the electrolytic product UO_2_ to study the deposition of different types and morphologies of particles on the electrode.

**Fig. 3:**
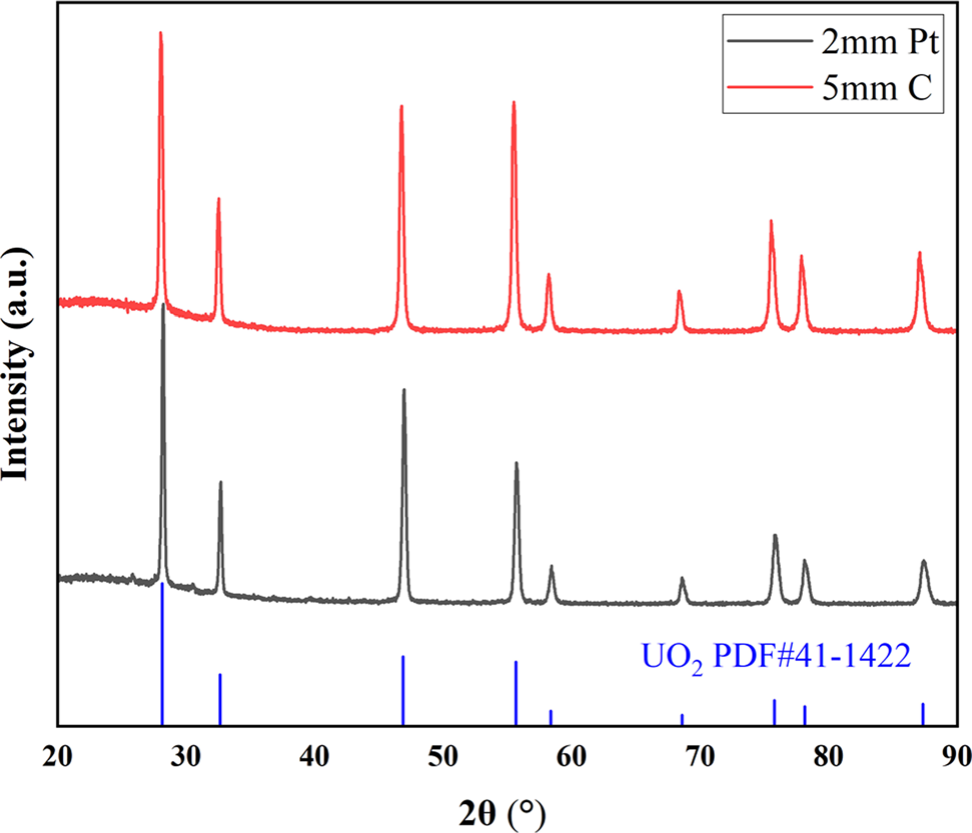
XRD spectra of products obtained by electrodeposition with different electrodes.

Fig. 3 shows the XRD spectrum of the UO_2_ product obtained when using *Φ* 5 mm graphite and *Φ* 2 mm platinum as the cathodes. All peaks correspond to the standard XRD pattern of UO_2_, and no other peaks are observed. This confirms that both material of electrodes can product pure UO_2_ in the electrodeposition reaction.

**Fig. 4:**
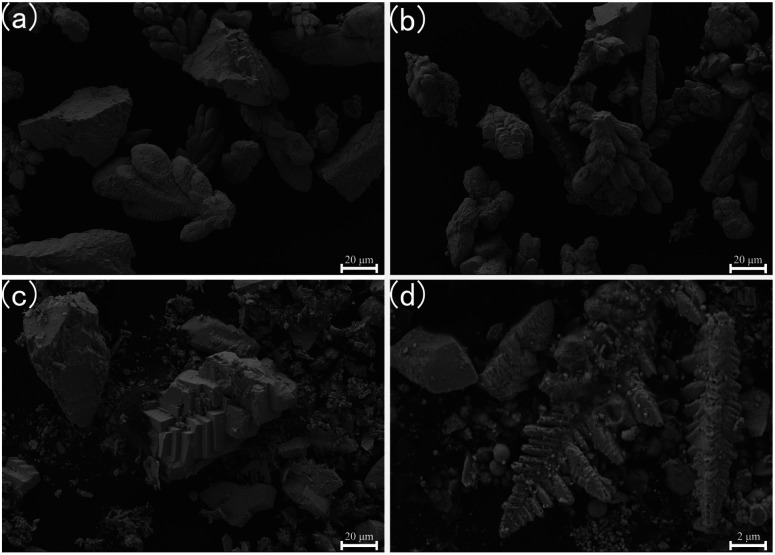
SEM image of the product after 2 h of electrolysis at 650 °C and −1.2 V; (a) and (b) deposition on *Φ* 5 mm graphite; (c) and (d) deposition on *Φ* 2 mm platinum.

The surface morphology of UO_2_ deposition formed under different cathodes and electrolysis voltages were analyzed using SEM-EDS. Fig. 4 presents the SEM images of the UO_2_ deposition obtained by electrolyzing at a constant potential of −1.2 V for 2 h in NaCl–2CsCl molten salt at 650 °C. When *Φ* 5 mm graphite was used as the cathode, UO_2_ were observed as dendritic particles (as shown in Fig. 4(a) and (b)). Conversely, when *Φ* 2 mm platinum was used as the cathode, UO_2_ products with blocky or powdered particles were obtained (as shown in Fig. 4(c) and (d)), this phenomenon was attributed to the different electrode surface roughness and solid electrolyte interface exhibited by graphite and platinum, which will affect the growth of dendrites.^28^ The particles size of both types of cathode depositions was in the micrometre range.

**Fig. 5:**
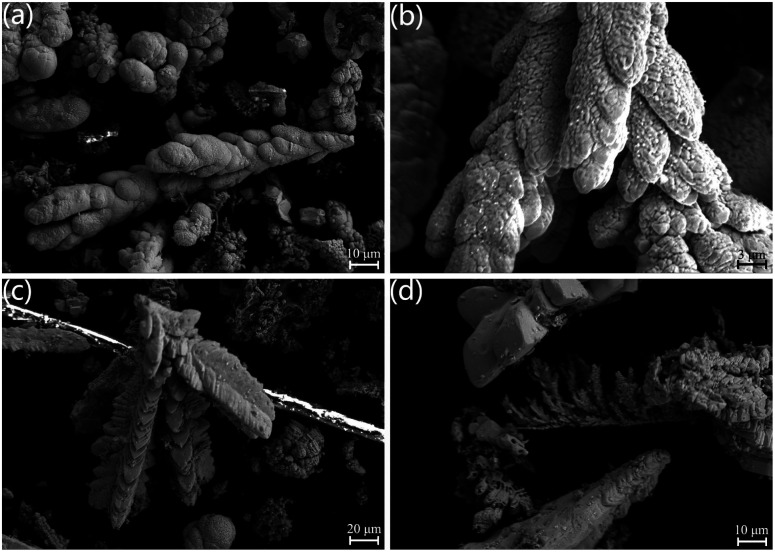
SEM image of the product obtained from electrolysis of 5 mm graphite at 650 °C for 5 h; (a) and (b) products deposited at −1.6 V; (c) and (d) products deposited at −0.8 V.

Fig. 5 illustrates the SEM images of the UO_2_ depositions obtained by electrolyzing at a constant voltage for 5 h using *Φ* 5 mm graphite as cathode in NaCl–2CsCl molten salt at 650 °C. Fig. 5(a) and (b) depict the products obtained at −1.6 V, exhibiting a morphology of large blocky dendritic particles. Fig. 5(c) and (d) show the products obtained at −0.8 V, consisting of smaller dendritic particles.

The uranium–oxygen ratio of the electrodeposited products under different conditions was determined by ICP-AES. A standard curve for uranium was prepared in the range of 0–10 ppm. As shown in Table 6 (with the same number as Tables 1–5), the mass of the electrodeposited products under different conditions was accurately weighed and dissolved in 5 mL of 6 mol L^−1^ HNO_3_. Then, 75 μL of the solution was taken and diluted with deionized water to a volume of 900 μL, resulting in a 0.5 mol L^−1^ HNO_3_ solution. This solution was further diluted 66.7 times with 0.5 mol L^−1^ HNO_3_. The concentration of uranium after dilution was measured, and the masses of uranium and oxygen in the products were calculated accordingly. The uranium–oxygen ratio was determined to be 1 : 2, confirming that the electrodeposited products were UO_2_.

**Table 5:** The effect of electrolysis voltage on electrodeposition reaction

No.	Cathode material and diameter (mm)	Temperature (°C)	Electrolysis time (h)	Uranium concentration in molten salt (U_3_O_8_ : molten salt)	Electrolysis voltage (V)	Recovery efficiency of UO_2_ (%)
11	Graphite *Φ* 5	650	9	1 : 100	*−0.8*	91
5	Graphite *Φ* 5	650	5	1 : 100	*−1.2*	95
**12**	**Graphite *Φ* 5**	**650**	**5**	**1** : **100**	***−1.6***	**97**

**Table 6:** Determination of uranium–oxygen ratio of products

No.	Mass of the sample/mg	Measured concentration of uranium/ppm	Mass of uranium in the sample/mg	Uranium–oxygen ratio
2	22.38	4.8755	19.50	0.46 ≈ 1 : 2
5	20.01	4.4464	17.79	0.54 ≈ 1 : 2
8	20.69	4.5441	18.18	0.49 ≈ 1 : 2
9	20.85	4.6974	18.79	0.61 ≈ 1 : 2
11	20.14	4.4071	17.63	0.47 ≈ 1 : 2
12	20.65	4.5109	18.04	0.46 ≈ 1 : 2

The uranium content in the molten salt after electrolysis under different conditions were determined using ICP-AES. A standard curve for uranium was prepared in the range of 0–2 ppm. The mass of the molten salt after electrolysis under different conditions were accurately weighed and dissolved in 10 mL of 0.5 mol L^−1^ HNO_3_. The uranium concentration after dilution was measured, and the mass and content of uranium in the molten salt after electrolysis were calculated accordingly. The results are shown in Table 7.

**Table 7:** The content of uranium in molten salt

No.	Mass of molten salt sample/mg	Measured concentration of uranium/ppm	Mass of uranium in molten salt sample/mg	The content of uranium in molten salt/%
2	71.51	0.9363	0.009363	0.010
5	63.82	0.4825	0.004825	0.008
8	41.62	0.3483	0.003483	0.008
12	57.36	0.3304	0.003304	0.006

### 2.5 Doping experiment

To investigate the influence of La, Ce, and Nd on uranium electrodeposition, these three elements were introduced into the reaction separately. Firstly, the doped La system was studied. Under the optimized conditions mentioned above, electrolysis was conducted at −1.2 V or −1.6 V, and the resulting products were characterized by XRD, ICP-AES, SEM-EDS, and XPS technologies to explore their effects on uranium recovery and decontamination factor.

**Fig. 6:**
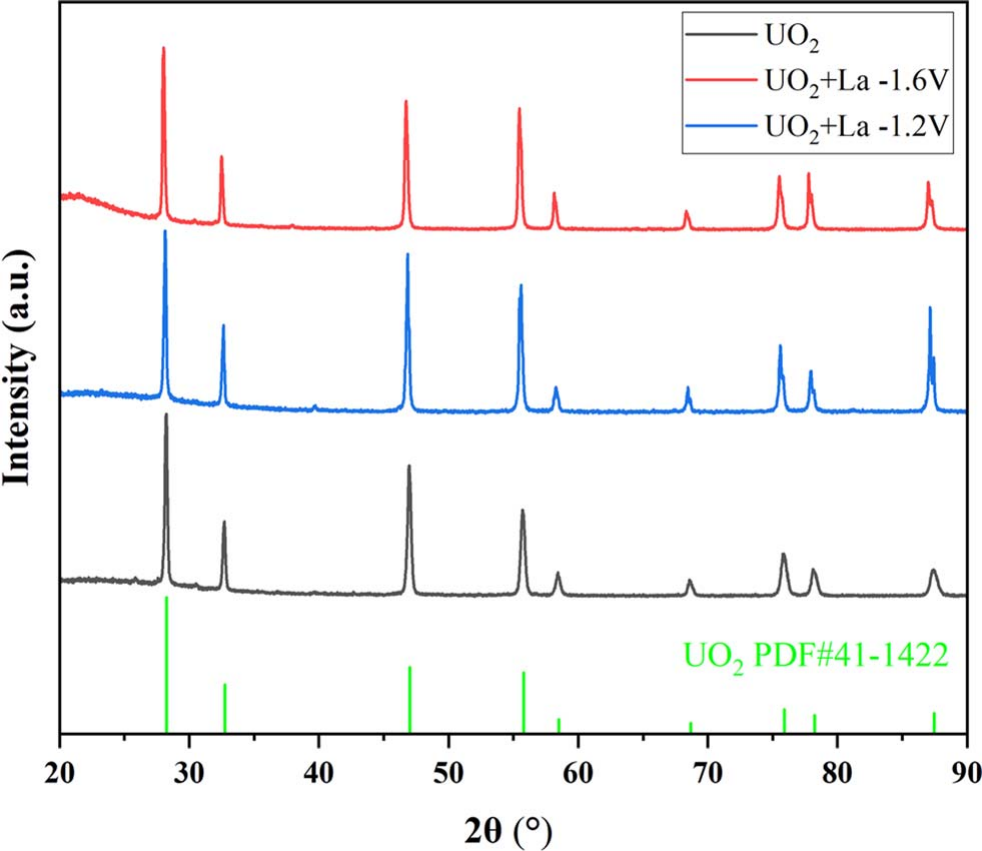
XRD spectra of products in doped La experiments.

As shown in Fig. 6, when La_2_O_3_ was introduced into U_3_O_8_ and electrolysis was performed at −1.2 V or −1.6 V after chlorination, the obtained UO_2_ products exhibited negligible impurity peaks in the XRD spectra, the data with black line represents the UO_2_ product obtained under optimal conditions. To analyze the content of the doped element in the product, ICP-AES measurements were performed on the products obtained at different electrolysis voltages. As shown in Table 8, compared to the undoped blank sample, the introduction of La had no significant effect on the recovery efficiency of product. After doping with La_2_O_3_ and electrolysis at −1.2 V or −1.6 V, the La content in the UO_2_ products was determined to be 0.2% and 0.3%, respectively. The decontamination factors of La were calculated as DF(La) = 119 at −1.2 V and DF(La) = 79 at −1.6 V, respectively. The results indicate that electrolysis at −1.2 V is more favorable for the separation of impurity elements. The decontamination factor (DF) is calculated using the following equation.
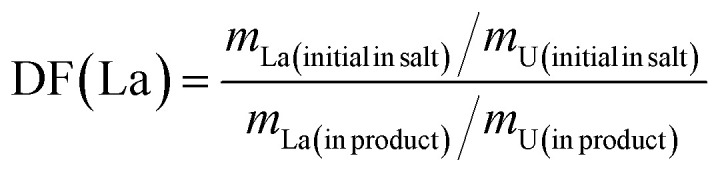


**Table 8:** The effect of doping elements on electrodeposition reaction

Entry	Doped element	Electrolysis voltages (V)	Recovery efficiency of UO_2_ (%)	Impurity metal content in the product (%)	Decontamination factor
1	La_2_O_3_	−1.2	95	0.2	DF(La) = 119
2	La_2_O_3_	−1.6	93	0.3	DF(La) = 79
3	CeO_2_	−1.2	88	1.2	DF(Ce) = 27
4	Nd_2_O_3_	−1.2	91	0.7	DF(Nd) = 57
5 (blank)	—	−1.2	95	—	—

**Fig. 7:**
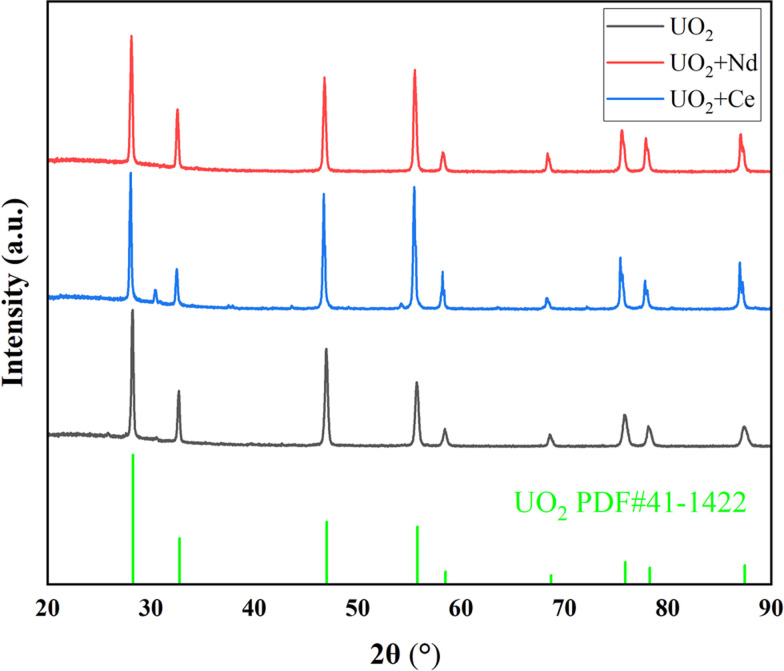
XRD spectra of products doped with Ce or Nd experiments.

On the other hand, under an electrolysis voltage of −1.2 V, electrodeposition reactions were carried out for the doped CeO_2_ or Nd_2_O_3_ systems, and the XRD spectra of the resulting products are shown in Fig. 7, the data with black line represents the UO_2_ product obtained under optimal conditions. In Fig. 7, the main product was UO_2_, but trace impurity peaks can be observed, which may be the generation of CeOCl.^29^ As shown in Table 8, the recovery efficiency of the UO_2_ products were 88% and 91% for Ce and Nd, respectively, and the Ce and Nd contents in the UO_2_ products were determined to be 1.2% and 0.7%, respectively. The calculated decontamination factors were DF(Ce) = 27 and DF(Nd) = 57. The results indicate that the system doped with lanthanide elements, under the given electrolysis conditions, the recovery efficiency of UO_2_ was slightly reduced. This could be attributed to the formation of a thin film of lanthanide elements on the surface of UO_2_, which inhibits the growth of UO_2_ crystals.^30–32^ As a result, some extremely fine UO_2_ particles were lost during centrifugation and washing processes, leading to a decrease in the recovery efficiency of UO_2_. Regarding the aspect of separation, the separation of La from uranium is relatively easier, while the separation of Ce from uranium is relatively poorer.

**Fig. 8:**
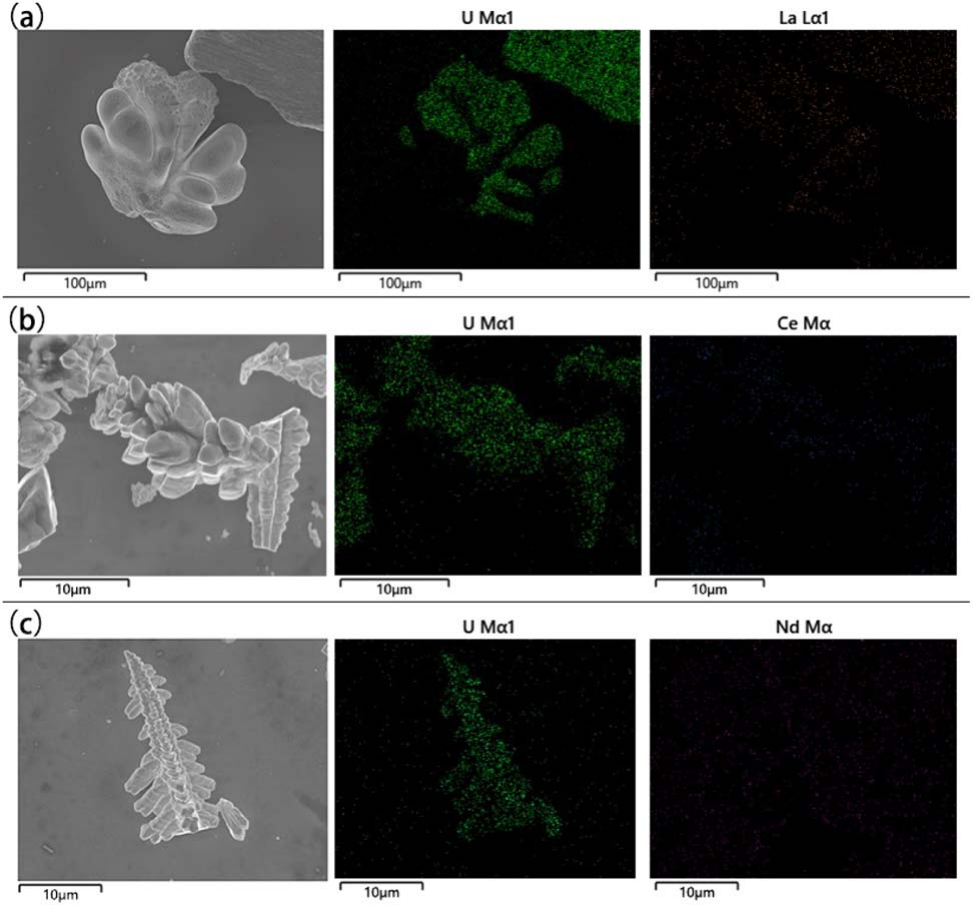
SEM-EDS spectra of different doping systems; (a) doping system with La; (b) doping system with Ce; (c) doping system with Nd.

The microstructures and elemental distribution of UO_2_ products obtained in the doping system were characterized by SEM-EDS respectively. As shown in Fig. 8, the electrolysis products of different doped elements were all relatively pure UO_2_, with the low content of impurity elements. The distribution of impurity elements was almost invisible in the EDS spectrum, which further proves the reliability of ICP-AES.

**Fig. 9:**
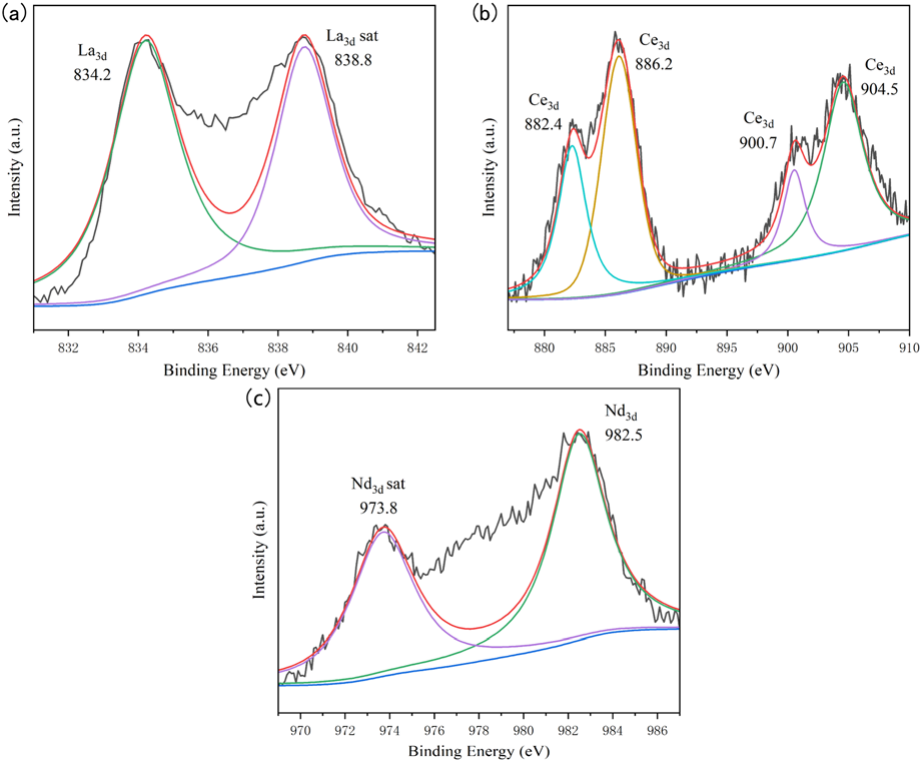
XPS spectra of different doping systems; (a) doping system with La; (b) doping system with Ce; (c) doping system with Nd.

The valence states of impurity elements in the UO_2_ product obtained from the doped system were analyzed by XPS, and the analysis data was fitted with peak deconvolution, as shown in Fig. 9. In the product of the doped La system, the binding energy of the La element was determined to be 834.2 eV, corresponding to the characteristic peak of La, and exist in the form of LaOCl.^33^ In the product of the doped Ce system, the binding energies of the Ce element were determined to be 882.4, 886.2, 900.7, and 904.5 eV, all corresponding to the characteristic peaks of Ce.^34^ When Nd was introduced, the binding energy of Nd element was 982.5 eV, which corresponding to the characteristic peak of Nd.^35,36^ Considering the high temperature and the presence of trace amounts of H_2_O and O_2_ in the chloride molten salt, during the electrolysis process, LaOCl, CeOCl, NdOCl could be formed and mixed with the UO_2_ product.^37,38^

## 3 Conclusions

In this study, electrodeposition technology was used to investigate the recovery of UO_2_ from spent oxide fuel. Dissolution of U_3_O_8_ was achieved through chlorination using NH_4_Cl. By means of a three-electrode system, the effects of cathode material and diameter, electrolysis temperature, electrolysis time, electrolysis voltage and uranium concentration in the molten salt on the electrolysis reaction had been explored. Consequently, increasing the cathode diameter, extending the electrolysis time, and appropriately increasing the reduction voltage can all contribute to improving the recovery efficiency of the electrolysis reaction. When using *Φ* 5 mm graphite as the cathode, *Φ* 6 mm graphite as the anode, and *Φ* 0.5 mm platinum as the reference electrode, the temperature of 650 °C, the mass ratio of U_3_O_8_ and molten salt is 1 : 100, pure UO_2_ was obtained by electrolytic reaction at −1.6 V for 5 h with a recovery efficiency of 97% and was characterized using XRD, SEM-EDS, ICP-AES and XPS.

Furthermore, doping experiments were conducted in this electrolyte system by introducing La, Ce, and Nd elements separately. Electrolysis voltage was performed at −1.2 V and −1.6 V, respectively. For introduction of La, the system exhibited better removal of La at −1.2 V, a decontamination factor as high as 119, which is superior to the −1.6 V (the decontamination factor decreased to 79). The system showed good purification effects for Nd, with a decontamination factor of 57 at an electrolysis voltage of −1.2 V. However, it was not effective in removing Ce, with a decontamination factor of only 27.

The further research work will be conducted on the removal of Ce in the future to achieve higher purification efficiency. Additionally, scaling up the research on electrodeposition and research on corrosion-resistant materials will be conducted to accelerate the research progress of electrolysis technology in the dry reprocessing of spent nuclear fuel.

## 4 Experimental section

### 4.1 Reagents and instruments

Sodium chloride (NaCl, anhydrous, 99.8% purity, Zancheng (Tianjin) Technology Co., Ltd.), cesium chloride (CsCl, anhydrous, 99.9% purity, Anhui Senrise Technology Co., Ltd.), and ammonium chloride (NH_4_Cl, anhydrous, GR grade, Aladdin Reagent (Shanghai) Co., Ltd.) were purchased. NaCl and CsCl were dried at 180 °C for over 12 h to minimize the amount of adsorbed water before use.

The phases composition was analyzed and tested by X-ray diffraction (XRD) diffractometer using Miniflex 600 (Rigaku Corp.) with Cu Kα radiation at 40 kV and 40 mA. Scanning electron microscopy (SEM, ZEISS GeminiSEM 360, Zeiss Germany Inc.) was used to investigate morphology and size of samples. Energy dispersive X-ray spectroscopy (EDS, AZtecone, Oxford Instruments) was used to analyze the products' composition with SEM. And the concentrations of the uranium were tested by inductively coupled plasma atomic emission spectrometer (ICP-AES, ARCOS FHS12, Spectro Scientific). The valence states of the products were analyzed by an X-ray photoelectron spectrometer (XPS, Escalab250Xi, ThermoFisher Scientific Inc.) equipped with an Al Kalpha source.

### 4.2 Experiment

#### 4.2.1 Oxidation of uranium dioxide

9.99 g of UO_2_ placed in a quartz crucible, heating at 650 °C for 5.5 h, with the formation of black powder.

#### 4.2.2 Chlorination reaction

##### 4.2.2.1 Chlorination reaction of pure U_3_O_8_

1.48 g NaCl, 8.52 g CsCl, 2 g NH_4_Cl, and 0.1 g U_3_O_8_ were grinded thoroughly, the mixture was transferred to a corundum crucible, and heat at 650 °C for 2 h to obtain an orange solution. Then cool to room temperature yielding a yellow block solid.

##### 4.2.2.2 Chlorination reaction of U_3_O_8_ with doping elements

Three systems were prepared with different doping elements:

(1) Doping with La element: 1.48 g NaCl, 8.52 g CsCl, 3 g NH_4_Cl, 0.1 g U_3_O_8_, and 49.7 mg La_2_O_3_ were accurately weighed, thoroughly mixed, and transferred to a corundum crucible. The crucible was then heated at 650 °C for 2 h and cooled to room temperature.

(2) Doping with Ce element: 1.48 g NaCl, 8.52 g CsCl, 3 g NH_4_Cl, 0.1 g U_3_O_8_ and 52.1 mg CeO_2_ were accurately weighed, thoroughly mixed, and transferred to a corundum crucible. The crucible was then heated at 650 °C for 2 h and cooled to room temperature.

(3) Doping with Nd element: 1.48 g NaCl, 8.52 g CsCl, 3 g NH_4_Cl, 0.1 g U_3_O_8_ and 49.5 mg Nd_2_O_3_ were accurately weighed, thoroughly mixed, and transferred to a corundum crucible. The crucible was then heated at 650 °C for 2 h and cooled to room temperature.

#### 4.2.3 Electrodeposition reaction

The electrodeposition reaction used a three-electrode system, all electrodes were submerged in deionized water and sonicated for 10 min, followed by cleaning them with ethanol. The platinum was polished with sandpaper and dried. At 650 °C, 0.1 g of U_3_O_8_ was chlorinated and dissolved in 10 g of molten salt (*n*(NaCl) : *n*(CsCl) = 1 : 2) in a corundum crucible with the method in Section 4.2.2. *Φ* 6 mm graphite was used as the anode, *Φ* 1 mm platinum was used as the cathode, *Φ* 0.5 mm platinum was used as the reference electrode, and electrolysis at −1.6 V for 5 h inside the glovebox. A black solid product was formed, most of which fell into the molten salt, leaving only a small amount of product on the electrodes. After cooling to room temperature, added 50 mL of deionized water, stirred for 20 min to dissolve all molten salt on the cathode or in the crucible, centrifuged at 3000 rpm for 2 min, pour off the supernatant, and then wash the solid with deionized water (50 mL × 2) and anhydrous ethanol (50 mL × 2). Dry the solid at 50 °C overnight to obtain a black solid powder.

## Author contributions

Pei Wu: conceptualization, methodology, validation, investigation, formal analysis, software, writing – original draft. Liqin Wang: conceptualization, data curation, software, writing – review & editing. Jinrui Wang: investigation, writing – original draft. Junhan Luo: methodology, resources, investigation. Yuexiang Lu: resources, methodology, formal analysis. Xiaopeng Song: methodology, supervision. Jilian Liu: investigation, formal analysis, conceptualization, writing – review& editing. Yongquan Qin: supervision, project administration, funding acquisition, writing – review& editing. Liudong Hou: conceptualization, resources, supervision, funding acquisition, project administration. Jing Ma: conceptualization, resources, supervision, project administration, funding acquisition, writing – review& editing.

## Conflicts of interest

There are no conflicts to declare.

## Supplementary Material
